# Partial Rectangular Metric Spaces and Fixed Point Theorems

**DOI:** 10.1155/2014/756298

**Published:** 2014-01-29

**Authors:** Satish Shukla

**Affiliations:** Department of Applied Mathematics, Shri Vaishnav Institute of Technology & Science Gram Baroli, Sanwer Road, Indore 453331, India

## Abstract

The purpose of this paper is to introduce the concept of partial rectangular metric spaces as a generalization of rectangular metric and partial metric spaces. Some properties of partial rectangular metric spaces and some fixed point results for quasitype contraction in partial rectangular metric spaces are proved. Some examples are given to illustrate the observed results.

## 1. Introduction

In 1906, the famous French mathematician Fréchet [1] introduced the concept of a metric space. After this, several mathematicians generalize the concept of metric space in different directions. Branciari [2] introduced a class of generalized (rectangular) metric spaces by replacing triangular inequality by similar one which involves four or more points instead of three and improved Banach contraction principle. On the other hand, Matthews [3] introduced the notion of partial metric space as a part of the study of denotational semantics of dataflow network. In this space, the usual metric is replaced by partial metric with an interesting property that the self-distance of any point of space may not be zero. Further, Matthews showed that the Banach contraction principle is valid in partial metric space and can be applied in program verification. Ćirić [4] introduced the quasicontractions in metric spaces and generalized the Banach contraction principle and several other fixed point theorems in metric spaces.

In this paper, we generalize the concept of rectangular metric space and extend the concept of partial metric space by introducing the partial rectangular metric space. A fixed point theorem for quasitype contraction is also proved in the partial rectangular metric space which generalizes several known results in metric, partial metric, and rectangular metric spaces. Results are illustrated by some examples.

First, we recall some definitions from partial metric and rectangular metric spaces (see [2, 3]).

**Definition 1**

A partial metric on a nonempty set *X* is a mapping *p* : *X* × *X* → ℝ such that, for all *x*, *y*, *z* ∈ *X*,

(P1) *p*(*x*, *y*) ≥ 0;
(P2) *x* = *y* if and only if *p*(*x*, *x*) = *p*(*x*, *y*) = *p*(*y*, *y*);
(P3) *p*(*x*, *x*) ≤ *p*(*x*, *y*);
(P4) *p*(*x*, *y*) = *p*(*y*, *x*);
(P5) *p*(*x*, *y*) ≤ *p*(*x*, *z*) + *p*(*z*, *y*) − *p*(*z*, *z*).

A partial metric space is a pair (*X*, *p*) such that *X* is a nonempty set and *p* is a partial metric on *X*.

**Definition 2**

Let *X* be a nonempty set and let *d* : *X* × *X* → ℝ be a mapping such that

(R1) 0 ≤ *d*(*x*, *y*), for all *x*, *y* ∈ *X* and *d*(*x*, *y*) = 0, if and only if *x* = *y*;
(R2) *d*(*x*, *y*) = *d*(*y*, *x*), for all *x*, *y* ∈ *X*;
(R3) *d*(*x*, *y*) ≤ *d*(*x*, *w*) + *d*(*w*, *z*) + *d*(*z*, *y*), for all *x*, *y* ∈ *X* and for all distinct points *w*, *z* ∈ *X* − {*x*, *y*} (rectangular property).

Then, *d* is called a rectangular metric on *X*, and (*X*, *d*) is called a rectangular metric space. A sequence {*x*_*n*_} in *X* is called convergent and converges to *x* ∈ *X*, if, for every *ɛ* > 0, there exists *n*_0_ ∈ *ℕ* such that *d*(*x*_*n*_, *x*) < *ɛ* for all *n* > *n*_0_. Sequence {*x*_*n*_} is called a Cauchy sequence if, for every *ɛ* > 0, there exists *n*_0_ ∈ *ℕ* such that *d*(*x*_*n*_, *x*_*m*_) < *ɛ*, for all *n*, *m* > *n*_0_. A rectangular metric space (*X*, *d*) is called complete, if every Cauchy sequence in *X* converges in *X*.

## 2. Partial Rectangular Metric Spaces

In this section, we define partial rectangular metric spaces and prove some properties of partial rectangular metric spaces.

**Definition 3**

Let *X* be a nonempty set and let *ρ* : *X* × *X* → ℝ be a mapping such that

(*ρ*1) *ρ*(*x*, *y*) ≥ 0, for all *x*, *y* ∈ *X*;
(*ρ*2) *x* = *y* if and only if *ρ*(*x*, *y*) = *ρ*(*x*, *x*) = *ρ*(*y*, *y*), for all *x*, *y* ∈ *X*;
(*ρ*3) *ρ*(*x*, *x*) ≤ *ρ*(*x*, *y*), for all *x*, *y* ∈ *X*;
(*ρ*4) *ρ*(*x*, *y*) = *ρ*(*y*, *x*), for all *x*, *y* ∈ *X*;
(*ρ*5) *ρ*(*x*, *y*) ≤ *ρ*(*x*, *w*) + *ρ*(*w*, *z*) + *ρ*(*z*, *y*) − *ρ*(*w*, *w*) − *ρ*(*z*, *z*), for all *x*, *y* ∈ *X* and for all distinct points *w*, *z* ∈ *X*∖{*x*, *y*}.

Then, *ρ* is called a partial rectangular metric on *X* and the pair (*X*, *ρ*) is called a partial rectangular metric space.

**Remark 4**

In a partial rectangular metric space (*X*, *ρ*), if *x*, *y* ∈ *X* and *ρ*(*x*, *y*) = 0, then *x* = *y* but the converse may not be true.

**Remark 5**

It is clear that every rectangular metric space is a partial rectangular metric space with zero self-distance. However, the converse of this fact need not hold.

**Example 6**

Let *X* = [0, *a*], *α* ≥ *a* ≥ 3 and define a mapping *ρ* : *X* × *X* → ℝ by

(1) $$\begin{array}{c} \rho \left(x,y\right)=\{\begin{array}{cc} x, & \text{if}\;\;x=y;\\ \frac{3\alpha +x+y}{2}, & \text{if}\;\;x,y\in \left\{1,2\right\},\;\;x\neq y;\\ \frac{\alpha +x+y}{2}, & \text{otherwise}. \end{array} \end{array}$$

Then, (*X*, *d*) is a partial rectangular metric space, but it is not a rectangular metric space, because *ρ*(*x*, *x*) ≠ 0, for all *x* > 0. Also, (*X*, *ρ*) is not a partial metric space because it lacks the property (P5). Indeed,

(2) ρ(1,2)=32(α+1)>ρ(1,3)+ρ(3,2)−ρ(3,3)=12(α+4)+12(α+5)−3=α+32.

Next proposition shows that every partial rectangular metric space induces a rectangular metric space.

**Proposition 7**

For each partial rectangular metric space (*X*, *ρ*), the pair (*X*, *ρ* ^*r*^) is a rectangular metric space, where

(3) $$\begin{array}{c} \rho^{r}\left(x,y\right)=2\rho \left(x,y\right)-\rho \left(x,x\right)-\rho \left(y,y\right)\;\forall x,y\in X. \end{array}$$

**Proof**

By definition of *ρ* and *ρ* ^*r*^, it is easy to verify that *ρ* ^*r*^ satisfies the properties (R1) and (R2). For (R3), let *x*, *y*, *z*, *w* ∈ *X*, where *w* and *z* are distinct and *w*, *z* ∈ *X*∖{*x*, *y*}; then we have

(4) ρr(x,y)=2ρ(x,y)−ρ(x,x)−ρ(y,y)≤2ρ(x,w)+2ρ(w,z)+2ρ(z,y) −2ρ(w,w)−2ρ(z,z)−ρ(x,x)−ρ(y,y)=[2ρ(x,w)−ρ(x,x)−ρ(w,w)] +[2ρ(w,z)−ρ(w,w)−ρ(z,z)] +[2ρ(z,y)−ρ(z,z)−ρ(y,y)]=ρr(x,w)+ρr(w,z)+ρr(z,y).

Thus, (*X*, *ρ* ^*r*^) is a rectangular metric space.

Here, (*X*, *ρ* ^*r*^) is called the induced rectangular metric space, and *ρ* ^*r*^ is the induced rectangular metric. In further discussion until specified, (*X*, *ρ* ^*r*^) will represent induced rectangular metric space.

Now, we show that a rectangular metric space with some restriction on it always induces a partial rectangular metric space.

**Proposition 8**

Let (*X*, *d*) be a rectangular metric space and there exists *x*_0_ ∈ *X* such that *d*(*x*, *x*_0_) ≤ *d*(*x*, *y*), for all distinct *x*, *y* ∈ *X*. Then, the pair (*X*, *ρ*) is a partial rectangular metric space, where

(5) $$\begin{array}{c} \rho \left(x,y\right)=\frac{1}{2}\left[d\left(x,y\right)+d\left(x,x_{0}\right)+d\left(y,x_{0}\right)\right]\;\forall x,y\in X. \end{array}$$

Also, the rectangular metric induced by *ρ* is the rectangular metric *d*.

**Proof**

The properties (*ρ*1) and (*ρ*4) follow by the definition of *ρ*. For (*ρ*2), note that *ρ*(*x*, *x*) = *d*(*x*, *x*_0_), for all *x* ∈ *X*; therefore, if *x*, *y* ∈ *X* and *ρ*(*x*, *y*) = *ρ*(*x*, *x*) = *ρ*(*y*, *y*), we have (1/2)[*d*(*x*, *y*) + *d*(*x*, *x*_0_) + *d*(*y*, *x*_0_)] = *d*(*x*, *x*_0_) = *d*(*y*, *x*_0_), which implies that *d*(*x*, *y*) = 0; that is, *x* = *y*.

By the choice of *x*_0_, the property (*ρ*3) follows immediately. For (*ρ*5), let *x*, *y*, *w*, *z* ∈ *X*,  *x* ≠ *y*,  *w*,  *z* ∈ *X*∖{*x*, *y*}. Then, by definition of *ρ*, we have

(6) ρ(x,y) =12[d(x,y)+d(x,x0)+d(y,x0)] ≤12[d(x,w)+d(w,z)+d(z,y)+d(x,x0)+d(y,x0)] =12[d(x,w)+d(x,x0)+d(w,x0)]  +12[d(w,z)+d(w,x0)+d(z,x0)]  +12[d(z,y)+d(z,x0)+d(y,x0)]  −d(w,x0)−d(z,x0) =ρ(x,w)+ρ(w,z)+ρ(z,y)−ρ(w,w)−ρ(z,z).

Thus, (*X*, *ρ*) is a partial rectangular metric space. Now, by the definition, it is easy to verify that *d* is the rectangular metric induced by *ρ*.

**Example 9**

Let *X* = {1/*n* : *n* ∈ *ℕ*} ∪ {0} and define *d* : *X* × *X* → ℝ by

(7) $$\begin{array}{c} d\left(x,y\right)=\{\begin{array}{cc} 0, & \text{if}\;\;x=y;\;\\ \frac{1}{n}, & \text{if}\;\;\left\{x,y\right\}=\left\{0,\frac{1}{n}\right\};\\ 1, & \text{if}\;\;x\neq y,\;\;x,y\in X\setminus \left\{0\right\}. \end{array} \end{array}$$

Then, (*X*, *d*) is a rectangular metric space. Note that *d*(*x*, 0) ≤ *d*(*x*, *y*) for all distinct *x*, *y* ∈ *X* and so *ρ* : *X* × *X* → ℝ defined by

(8) ρ(x,y)=12[d(x,y)+d(x,0)+d(y,0)]={x,if  x=y;1n,if  {x,y}={0,1n};12[1+x+y],if  x≠y,  x,y∈X∖{0},

is a partial rectangular metric on *X*,  (*X*, *ρ*) is a partial rectangular metric space, and *d* is the rectangular metric induced by *ρ*.

The proof of following proposition is straightforward and, by using it, one can obtain some more examples of partial rectangular metric space.

**Proposition 10**

For any rectangular metric space (*X*, *d*) and constant *α* ≥ 0, the pair (*X*, *ρ*) is a partial rectangular metric space, where

(9) $$\begin{array}{c} \rho \left(x,y\right)=d\left(x,y\right)+\alpha \;\forall x,y\in X. \end{array}$$

Now, we define the convergence of a sequence and Cauchy sequence in partial rectangular metric spaces.

**Definition 11**

Let (*X*, *ρ*) be a partial rectangular metric space, {*x*_*n*_} a sequence in *X*, and *x* ∈ *X*. Then,

(i) the sequence {*x*_*n*_} is said to be convergent and converges to *x* ∈ *X*, if lim⁡_*n*→*∞*_ *ρ*(*x*_*n*_, *x*) = *ρ*(*x*, *x*);
(ii) the sequence {*x*_*n*_} is said to be Cauchy in (*X*, *ρ*), if lim⁡_*n*,*m*→*∞*_ *ρ*(*x*_*n*_, *x*_*m*_) exists and is finite;
(iii) (*X*, *ρ*) is said to be a complete partial rectangular metric space, if, for every Cauchy sequence {*x*_*n*_} in *X*, there exists *x* ∈ *X* such that
  (10) $$\begin{array}{c} \underset{n,m\to \infty }{\lim}\rho \left(x_{n},x_{m}\right)=\underset{n\to \infty }{\lim}\rho \left(x_{n},x\right)=\rho \left(x,x\right). \end{array}$$

Note that in a partial rectangular metric space the limit of convergent sequence may not be unique.

**Example 12**

Let *X* = {1/*n* : *n* ∈ *ℕ*} ∪ {0,2},  *α* ≥ 0 be a constant and define *ρ* : *X* × *X* → ℝ by

(11) ρ(x,y) ={α,if  x=y;1n+α,if  x∈{0,2},  y=1n  or  y∈{0,2},  x=1n;  1+α,otherwise.

Then, (*X*, *ρ*) is a partial rectangular metric space. Consider the sequence {*x*_*n*_} in *X*, where *x*_*n*_ = 1/*n*. Then, lim⁡_*n*→*∞*_ *ρ*(*x*_*n*_, 0) = lim⁡_*n*→*∞*_[1/*n* + *α*] = *α* = *ρ*(0,0) and lim⁡_*n*→*∞*_ *ρ*(*x*_*n*_, 2) = lim⁡_*n*→*∞*_[1/*n* + *α*] = *α* = *ρ*(2,2). Therefore, {*x*_*n*_} has two limits, namely, 0 and 2.

**Lemma 13**

Let (*X*, *ρ*) be a partial rectangular metric space and let {*x*_*n*_} be a sequence in *X*. Then, the sequence {*x*_*n*_} converges in (*X*, *ρ* ^*r*^) and converges to *x* ∈ *X*; that is, lim⁡_*n*→*∞*_ *ρ* ^*r*^(*x*_*n*_, *x*) = 0, if and only if lim⁡_*n*→*∞*_ *ρ*(*x*_*n*_, *x*) = lim⁡_*n*→*∞*_ *ρ*(*x*_*n*_, *x*_*n*_) = *ρ*(*x*, *x*).

**Proof**

Note that

(12) lim⁡n→∞ρr(xn,x)=0  ⟺lim⁡n→∞[2ρ(xn,x)−ρ(xn,xn)−ρ(x,x)]=0  ⟺lim⁡n→∞[ρ(xn,x)−ρ(x,x)]=0,       lim⁡n→∞[ρ(xn,x)−ρ(xn,xn)]=0  ⟺lim⁡n→∞ρ(xn,x)=lim⁡n→∞ρ(xn,xn)=ρ(x,x),

which proves the result.

**Lemma 14**

Let (*X*, *ρ*) be a partial rectangular metric space and let {*x*_*n*_} be a sequence in *X*. Then, the sequence {*x*_*n*_} is a Cauchy sequence in (*X*, *ρ*) if and only if it is a Cauchy sequence in (*X*, *ρ* ^*r*^).

**Proof**

Let {*x*_*n*_} be a Cauchy sequence in (*X*, *ρ* ^*r*^); that is,

(13) lim⁡n,m→∞ρr(xn,xm)  =lim⁡n,m→∞[2ρ(xn,xm)−ρ(xn,xn)−ρ(xm,xm)]=0.

Note that |*ρ*(*x*_*n*_, *x*_*n*_) − *ρ*(*x*_*m*_, *x*_*m*_)|≤*ρ* ^*r*^(*x*_*n*_, *x*_*m*_), for all *n*, *m* ∈ *ℕ*; therefore, the sequence {*ρ*(*x*_*n*_, *x*_*n*_)} is a Cauchy sequence in ℝ^+^ and so we have lim⁡_*n*→*∞*_ *ρ*(*x*_*n*_, *x*_*n*_) ∈ ℝ^+^. Therefore, it follows from (13) that lim⁡_*n*,*m*→*∞*_ *ρ*(*x*_*n*_, *x*_*m*_) ∈ ℝ^+^.

Conversely, if {*x*_*n*_} is a Cauchy sequence in (*X*, *ρ*), that is, lim⁡_*n*,*m*→*∞*_ *ρ*(*x*_*n*_, *x*_*m*_) ∈ ℝ^+^, then again lim⁡_*n*→*∞*_ *ρ*(*x*_*n*_, *x*_*n*_) ∈ ℝ^+^ and by definition lim⁡_*n*,*m*→*∞*_ *ρ* ^*r*^(*x*_*n*_, *x*_*m*_) = 0.

The proof of the following lemma follows from Lemmas 13 and 14.

**Lemma 15**

A partial rectangular metric space is complete, if and only if its induced rectangular metric space is complete.

## 3. Fixed Point Theorems

In this section, some fixed point theorems in partial rectangular metric spaces are proved.

Let (*X*, *ρ*) be a partial rectangular metric space and let *T* : *X* → *X* be a mapping. For *A* ⊂ *X*, we denote the diameter of *A* by *δ*[*A*] and

(14) $$\begin{array}{c} \delta \left[A\right]=\sup\left\{\rho \left(x,y\right):x,y\in X\right\}. \end{array}$$

Note that, apart from rectangular metric space, if *A* = {*x*} ⊂ *X*, then *δ*[*A*] need not be zero, but *δ*[*A*] = *ρ*(*x*, *x*).

For each *x* ∈ *X*, we define the orbit of *T* by

(15) $$\begin{array}{c} O\left(x,n\right)=\left\{x,Tx,T^{2}x,\ldots ,T^{n}x\right\},\;n\in \mathbb{N},\\ O\left(x,\infty \right)=\left\{x,Tx,T^{2}x,\ldots \right\}. \end{array}$$

**Definition 16**

Let (*X*, *ρ*) be a complete partial rectangular metric space and let *T* : *X* → *X* be a mapping. Then, *T* is called a quasicontraction on *X* with constant *λ*, if it satisfies the following property:

(16) ρ(Tx,Ty)  ≤λmax⁡{ρ(x,y),ρ(x,Tx),      ρ(y,Ty),ρ(x,Ty),ρ(y,Tx)},

for all *x*, *y* ∈ *X*, where *λ* ∈ [0,1).

**Lemma 17**

Let (*X*, *ρ*) be a partial rectangular metric space, let *T* : *X* → *X* be a quasicontraction on *X* with constant *λ*, and let *n* be a positive integer. Then, for each *x* ∈ *X* and all *i*, *j* ∈ {1,2,…, *n*}, one has *ρ*(*T* ^*i*^ *x*, *T* ^*j*^ *x*) ≤ *λδ*[*O*(*x*, *n*)]. Furthermore there exists a positive integer *k* such that *k* ∈ {1,2,…, *n*} and *δ*[*O*(*x*, *n*)] = *ρ*(*x*, *T* ^*k*^ *x*).

**Proof**

Suppose *i*, *j* ∈ {1,2,…, *n*} then *T* ^*i*−1^ *x*, *T* ^*j*−1^ *x*, *T* ^*i*^ *x*, *T* ^*j*^ *x* ∈ *O*(*x*, *n*) and so, by (16), we have

(17) ρ(Tix,Tjx)=ρ(TTi−1x,TTj−1x)≤λmax⁡{ρ(Ti−1x,Tj−1x),ρ(Ti−1x,TTi−1x),     ρ(Tj−1x,TTj−1x),ρ(Ti−1x,TTj−1x),     ρ(Tj−1x,TTi−1x)}=λmax⁡{ρ(Ti−1x,Tj−1x),ρ(Ti−1x,Tix),ρ(Tj−1x,Tjx),    ρ(Ti−1x,Tjx),ρ(Tj−1x,Tix)}≤λδ[O(x,n)].

As *λ* ∈ [0,1), we have *ρ*(*T* ^*i*^ *x*, *T* ^*j*^ *x*) < *δ*[*O*(*x*, *n*)] and so, by the definition of orbit of *T* and the diameter *δ* of a set, we must have *δ*[*O*(*x*, *n*)] = *ρ*(*x*, *T* ^*k*^ *x*), where *k* ∈ {1,2,…, *n*}.

The next lemma shows that the orbit of a quasicontraction is necessarily bounded.

**Lemma 18**

Suppose that all the conditions of Lemma 17 are satisfied; then

(18) $$\begin{array}{c} \delta \left[O\left(x,\infty \right)\right]\leq \frac{1}{1-\lambda }\max\left\{\rho \left(x,Tx\right),\rho \left(x,T^{2}x\right)\right\} \end{array}$$

holds, for all *x* ∈ *X*.

**Proof**

Let *x* ∈ *X* be arbitrary. Note that {*δ*[*O*(*x*, *n*)]} is a nondecreasing sequence of real numbers, so

(19) $$\begin{array}{c} \delta \left[O\left(x,\infty \right)\right]=\sup\left\{\delta \left[O\left(x,n\right)\right]:n\in \mathbb{N}\right\}. \end{array}$$

Therefore, it is sufficient to show that

(20) δ[O(x,n)] ≤11−λmax⁡{ρ(x,Tx),ρ(x,T2x)} ∀x∈X,  n∈ℕ.

Now, (20) holds trivially, if *n* = 1. If *n* = 2, then from (*ρ*3) and (16), we have

(21) ρ(Tx,T2x) =ρ(Tx,TTx) ≤λmax⁡{ρ(x,Tx),ρ(x,Tx),     ρ(Tx,T2x),ρ(x,T2x),ρ(Tx,Tx)} =λmax⁡{ρ(x,Tx),ρ(Tx,T2x),ρ(x,T2x)}.

Since *λ* ∈ [0,1), we must have *ρ*(*Tx*, *T* ^2^ *x*) ≤ max⁡{*ρ*(*x*, *Tx*), *ρ*(*x*, *T* ^2^ *x*)}. Also, since *ρ*(*z*, *z*) ≤ *ρ*(*z*, *y*), for all *y*, *z* ∈ *X*, therefore (20) holds for *n* = 2.

Suppose *n* ≥ 3, then, by Lemma 17, we have *δ*[*O*(*x*, *n*)] = *ρ*(*x*, *T* ^*k*^ *x*), where *k* ∈ {1,2,…, *n*}. If *k* = 1 or *k* = 2, we have finished. Suppose *k* ≥ 3. If *Tx* = *x* or *T* ^2^ *x* = *x* or *T* ^2^ *x* = *Tx*, then we have *T* ^*k*^ *x* ∈ {*x*, *Tx*} and (20) holds. Similarly, if *T* ^*k*^ *x* = *T* ^2^ *x*, then (20) holds. Therefore, we can assume that *x*, *Tx*, *T* ^2^ *x*, *T* ^*k*^ *x* all are distinct elements of *X*. As *ρ*(*Tx*, *T* ^2^ *x*) ≤ max⁡{*ρ*(*x*, *Tx*), *ρ*(*x*, *T* ^2^ *x*)}, therefore, from (*ρ*5), (16), (21), and Lemma 17, we obtain

(22) ρ(x,Tkx)≤ρ(x,Tx)+ρ(Tx,T2x) +ρ(T2x,Tkx)−ρ(Tx,Tx)−ρ(T2x,T2x)≤ρ(x,Tx)+λmax⁡{ρ(x,Tx),ρ(x,T2x)} +ρ(TTx,Tk−1Tx)≤(1+λ)max⁡{ρ(x,Tx),ρ(x,T2x)} +λδ[O(Tx,k−1)].

Again, by Lemma 17, for some *l* ∈ {1,2,…, *k* − 1}, we have *δ*[*O*(*Tx*, *k* − 1)] = *ρ*(*Tx*, *T* ^*l*^ *Tx*) ≤ *λδ*[*O*(*x*, *l* + 1)] ≤ *λδ*[*O*(*x*, *n*)] = *λρ*(*x*, *T* ^*k*^ *x*); therefore, it follows from the above inequality that

(23) $$\begin{array}{c} \left(1-\lambda^{2}\right)\rho \left(x,T^{k}x\right)\leq \left(1+\lambda \right)\max\left\{\rho \left(x,Tx\right),\rho \left(x,T^{2}x\right)\right\}; \end{array}$$

that is,

(24) δ[O(x,n)]=ρ(x,Tkx)≤11−λmax⁡{ρ(x,Tx),ρ(x,T2x)},

and the result follows.

In the next theorem, existence and uniqueness of fixed point of quasicontraction in complete partial rectangular metric spaces are proved.

**Theorem 19**

Let (*X*, *ρ*) be a complete partial rectangular metric space and let *T* : *X* → *X* be a quasicontraction on *X* with constant *λ*. Then, *T* has a unique fixed point *u* ∈ *X* and *ρ*(*u*, *u*) = 0.

**Proof**

Let us first show that, if fixed point of *T* exists, then it is unique. Let *u*, *v* ∈ *X* be two distinct fixed points of *T*; that is, *Tu* = *u*, *Tv* = *v* and *ρ*(*u*, *v*) > 0. Then, from (16), we have

(25) ρ(u,v)=ρ(Tu,Tv)≤λmax⁡{ρ(u,v),ρ(u,Tu),    ρ(v,Tv),ρ(u,Tv),ρ(v,Tu)}=λmax⁡{ρ(u,v),ρ(u,u),ρ(v,v),ρ(u,v),ρ(v,u)}=λρ(u,v)<ρ(u,v).

This contradiction shows that *ρ*(*u*, *v*) = 0; that is, *u* = *v*. Thus, if fixed point of *T* exists, then it is unique. Further, if *u* ∈ *X* is a fixed point of *T* and *ρ*(*u*, *u*) > 0, then, from (16), we have

(26) ρ(u,u)=ρ(Tu,Tv)≤λmax⁡{ρ(u,u),ρ(u,Tu),    ρ(u,Tu),ρ(u,Tu),ρ(u,Tu)}=λρ(u,u)<ρ(u,u).

Again, this contradiction shows that *ρ*(*u*, *u*) = 0. So, if *u* is a fixed point of *T*, then *ρ*(*u*, *u*) = 0.

Now, we prove existence of fixed point of *T*. For arbitrary *x* ∈ *X*, we will show that the iterative sequence {*T* ^*n*^ *x*} is a Cauchy sequence. Let *m*, *n* ∈ *ℕ* with *m* > *n*. Then, using Lemma 17, we have

(27) ρ(Tnx,Tmx)=ρ(TTn−1x,Tm−n+1Tn−1x)≤λδ[O(Tn−1x,m−n+1)].

Again, by Lemma 17, there exists *k* ∈ {1,2,…, *m* − *n* + 1} such that *δ*[*O*(*T* ^*n*−1^ *x*, *m* − *n* + 1)] = *ρ*(*T* ^*n*−1^ *x*, *T* ^*k*^ *T* ^*n*−1^ *x*); therefore, using Lemma 17, it follows from the above inequality that

(28) ρ(Tnx,Tmx)  ≤λρ(Tn−1x,TkTn−1x)  =λρ(TTn−2x,Tk+1Tn−2x)  ≤λ2δ[O(Tn−2x,m−n+2)]  (as  k∈{1,2,…,m−n+1}).

By repetition of this process, we obtain

(29) $$\begin{array}{c} \rho \left(T^{n}x,T^{m}x\right)\leq \lambda^{n}\delta \left[O\left(x,m\right)\right], \end{array}$$

which together with Lemma 18 yields

(30) ρ(Tnx,Tmx)  ≤λn1−λmax⁡{ρ(x,Tx),ρ(x,T2x)} ∀n∈ℕ.

As *λ* ∈ [0,1), it follows from (30) that

(31) $$\begin{array}{c} \underset{n\to \infty }{\lim}\rho \left(T^{n}x,T^{m}x\right)=0. \end{array}$$

Therefore, {*T* ^*n*^ *x*} is a Cauchy sequence. By completeness of space (*X*, *ρ*), there exists *u* ∈ *X* such that

(32) $$\begin{array}{c} \underset{n,m\to \infty }{\lim}\rho \left(T^{n}x,T^{m}x\right)=\underset{n\to \infty }{\lim}\rho \left(T^{n}x,u\right)=\rho \left(u,u\right)=0. \end{array}$$

We will show that *u* is the fixed point of *T*. Suppose *ρ*(*Tu*, *u*) > 0. Without loss of generality, we can assume that *T* ^*n*^ *x* ≠ *T* ^*n*+1^ *x*, for all *n* ∈ *ℕ*, also, there exists *n*_0_ ∈ *ℕ* such that *T* ^*n*^ *x* ∉ {*u*, *Tu*}, for all *n* > *n*_0_. Therefore, it follows from (*ρ*5) that

(33) ρ(Tu,u)≤ρ(Tu,Tn+1x)+ρ(Tn+1x,Tnx)+ρ(Tnx,u) ∀n>n0.

Using (16), we have

(34) ρ(Tu,Tn+1x)  ≤λmax⁡{ρ(u,Tnx),ρ(u,Tu),      ρ(Tnx,TTnx),ρ(u,TTnx),ρ(Tnx,Tu)}  =λmax⁡{ρ(u,Tnx),ρ(u,Tu),ρ(Tnx,Tn+1x),      ρ(u,Tn+1x),ρ(Tnx,Tu)}.

For sufficiently large *n*, from (32) and the above inequality, we have

(35) $$\begin{array}{c} \rho \left(Tu,T^{n+1}x\right)\leq \lambda \max\left\{\rho \left(u,Tu\right),\rho \left(T^{n}x,Tu\right)\right\}. \end{array}$$

If *ρ*(*T* ^*n*^ *x*, *Tu*) ≤ *ρ*(*u*, *Tu*), then, from (32), (33), and (35), we obtain *ρ*(*Tu*, *u*) = 0. If *ρ*(*u*, *Tu*) ≤ *ρ*(*T* ^*n*^ *x*, *Tu*), then, for sufficiently large *n*, we have

(36) ρ(Tu,Tn+1x)  ≤λρ(Tnx,Tu)  ≤λ[ρ(Tnx,u)+ρ(u,Tn+1x)+ρ(Tn+1x,Tu)].

Again from (32), (33), (35), and (36), we obtain *ρ*(*Tu*, *u*) = 0. Therefore, we must have *Tu* = *u*. Thus, *u* is the unique fixed point of *T*.

We illustrate the above result by the following example, which additionally shows that a quasicontraction in a partial rectangular metric space may not be a quasicontraction in the induced rectangular metric space and, therefore, it shows that our generalization is proper.

**Example 20**

Let *X* = {0,1, 2,3, 4,5} and define *ρ* : *X* × *X* → ℝ by

(37) $$\begin{array}{c} \rho \left(x,y\right)=\{\begin{array}{cc} x, & \text{if}\;\;x=y;\\ \frac{15+x+y}{2}, & \text{if}\;\;x,y\in \left\{1,2\right\},\;\;x\neq y;\\ \frac{5+x+y}{2}, & \text{otherwise}.\; \end{array} \end{array}$$

Then, (*X*, *ρ*) is a complete partial rectangular metric space. Since, for all *x* ∈ *X*, *x* > 0,  *ρ*(*x*, *x*) = *x* > 0, therefore (*X*, *ρ*) is not a rectangular metric space. Also, (*X*, *ρ*) is not a partial metric space because it lacks the property (P5). Indeed,

(38) $$\begin{array}{c} \rho \left(1,2\right)=9>\rho \left(1,3\right)+\rho \left(3,2\right)-\rho \left(3,3\right)=\frac{9}{2}+5-3=\frac{13}{2}. \end{array}$$

Define a mapping *T* : *X* → *X* by

(39) $$\begin{array}{c} T0=T1=T2=0,\;\;T3=2,\\ T4=0,\;\;T5=4. \end{array}$$

Then, by a careful calculation, one can see that *T* is a quasicontraction with constant *λ* ∈ [11/14, 1). All the conditions of Theorem 19 are satisfied and *T* has a unique fixed point *u* = 0. Note that the rectangular metric induced by *ρ* is given by

(40) $$\begin{array}{c} \rho^{r}\left(x,y\right)=\{\begin{array}{cc} 0, & \text{if}\;\;x=y;\\ 15, & \text{if}\;\;x,y\in \left\{1,2\right\},\;\;x\neq y;\\ 5, & \text{otherwise}. \end{array} \end{array}$$

Now, it is easy to see that *T* is not a quasicontraction with respect to *ρ* ^*r*^. Indeed, for *x* = 4, *y* = 5, we have *ρ* ^*r*^(*Tx*, *Ty*) = 5 and *ρ* ^*r*^(*x*, *y*) = 5, *ρ* ^*r*^(*x*, *Tx*) = 5, *ρ* ^*r*^(*y*, *Ty*) = 5, *ρ* ^*r*^(*x*, *Ty*) = 0, and  *ρ* ^*r*^(*y*, *Tx*) = 5. Therefore, there exists no *λ* ∈ [0,1) such that

(41) ρr(Tx,Ty)  ≤λmax⁡{ρr(x,y),ρr(x,Tx),ρr(y,Ty),      ρr(x,Ty),ρr(y,Tx)},

for all *x*, *y* ∈ *X*. Thus, *T* is not a quasicontraction in the induced rectangular metric space.

**Remark 21**

Note that, in the above example, the mapping *T* is a Banach contraction in the partial rectangular metric space (*X*, *ρ*); that is, *ρ*(*Tx*, *Ty*) ≤ *λρ*(*x*, *y*), for all *x*, *y* ∈ *X* with *λ* ∈ [9/10, 1), while it is not even a quasicontraction in the induced rectangular metric space (*X*, *ρ* ^*r*^). Also (*X*, *ρ*) is not a partial metric space; therefore, the results of Branciari [2] and Matthews [3] are not applicable. Thus, this example also shows that the class of Banach contractions in a partial rectangular metric space is more wider than that in rectangular metric spaces.

The following corollaries generalize the Banach, Kannan, Reich, Chatterjea, and Hardy-Rogers fixed point results (for details, see [5]) in partial rectangular metric spaces.

**Corollary 22 (Banach type)**

Let (*X*, *ρ*) be a complete partial rectangular metric space and let *T* : *X* → *X* be a mapping. Suppose that the following condition is satisfied:

(42) $$\begin{array}{c} \rho \left(Tx,Ty\right)\leq \lambda \rho \left(x,y\right)\;\forall x,y\in X, \end{array}$$

where *λ* ∈ [0,1). Then, *T* has a unique fixed point *u* ∈ *X* and *ρ*(*u*, *u*) = 0.

**Corollary 23 (Kannan type)**

Let (*X*, *ρ*) be a complete partial rectangular metric space and let *T* : *X* → *X* be a mapping. Suppose that the following condition is satisfied:

(43) $$\begin{array}{c} \rho \left(Tx,Ty\right)\leq \alpha \left[\rho \left(x,Tx\right)+\rho \left(y,Ty\right)\right], \end{array}$$

for all *x*, *y* ∈ *X*, where *α* ∈ [0, 1/2). Then, *T* has a unique fixed point *u* ∈ *X* and *ρ*(*u*, *u*) = 0.

**Corollary 24 (Reich type)**

Let (*X*, *ρ*) be a complete partial rectangular metric space and let *T* : *X* → *X* be a mapping. Suppose that the following condition is satisfied:

(44) $$\begin{array}{c} \rho \left(Tx,Ty\right)\leq \alpha \rho \left(x,y\right)+\beta \rho \left(x,Tx\right)+\gamma \rho \left(y,Ty\right), \end{array}$$

for all *x*, *y* ∈ *X*, where *α*, *β*, and *γ* are nonnegative constants such that *α* + *β* + *γ* < 1. Then, *T* has a unique fixed point *u* ∈ *X* and *ρ*(*u*, *u*) = 0.

**Corollary 25 (Chatterjea type)**

Let (*X*, *ρ*) be a complete partial rectangular metric space and let *T* : *X* → *X* be a mapping. Suppose that the following condition is satisfied:

(45) $$\begin{array}{c} \rho \left(Tx,Ty\right)\leq \alpha \left[\rho \left(x,Ty\right)+\rho \left(y,Tx\right)\right], \end{array}$$

for all *x*, *y* ∈ *X*, where *α* ∈ [0, 1/2). Then, *T* has a unique fixed point *u* ∈ *X* and *ρ*(*u*, *u*) = 0.

**Corollary 26 (Hardy-Rogers type)**

Let (*X*, *ρ*) be a complete partial rectangular metric space and let *T* : *X* → *X* be a mapping. Suppose that the following condition is satisfied:

(46) ρ(Tx,Ty)≤αρ(x,y)+βρ(x,Tx)       +γρ(y,Ty)+μρ(x,Ty)+λρ(y,Tx),

for all *x*, *y* ∈ *X*, where *α*, *β*, *γ*, *μ*, and *λ* are nonnegative constants such that *α* + *β* + *γ* + *μ* + *λ* < 1. Then, *T* has a unique fixed point *u* ∈ *X* and *ρ*(*u*, *u*) = 0.

**Corollary 27**

Let (*X*, *ρ*) be a complete partial rectangular metric space and let *T* : *X* → *X* be a mapping. Suppose that for some positive integer *n*, the following condition is satisfied:

(47) ρ(Tnx,Tny)  ≤λmax⁡{ρ(x,y),ρ(x,Tnx),ρ(y,Tny),      ρ(x,Tny),ρ(y,Tnx)},

for all *x*, *y* ∈ *X*, where *λ* ∈ [0,1). Then, *T* has a unique fixed point *u* ∈ *X* and *ρ*(*u*, *u*) = 0.

**Proof**

We note that *T* ^*n*^ satisfies the condition (16) of Theorem 19; therefore, *T* ^*n*^ has a unique fixed point *u* ∈ *X* and *ρ*(*u*, *u*) = 0. Now, *T* ^*n*^ *Tu* = *TT* ^*n*^ *u* = *Tu*; therefore, *Tu* is another fixed point of *T* ^*n*^ and, by uniqueness, we have *Tu* = *u*. Thus, *u* is a fixed point of *T*. Since every fixed point of *T* is also a fixed point of *T* ^*n*^, the fixed point of  *T* is unique.
